# Refractory Chronic Spontaneous Urticaria Treated With Omalizumab in an Adolescent With Common Variable Immunodeficiency

**DOI:** 10.3389/fimmu.2019.01700

**Published:** 2019-07-17

**Authors:** Pasquale Comberiati, Giorgio Costagliola, Niccolò Carli, Annalisa Legitimo, Sofia D'Elios, Rita Consolini, Diego G. Peroni

**Affiliations:** ^1^Section of Paediatrics, Department of Clinical and Experimental Medicine, University of Pisa, Pisa, Italy; ^2^Department of Clinical Immunology and Allergology, I.M. Sechenov First Moscow State Medical University, Moscow, Russia

**Keywords:** chronic urticaria, common variable immunodeficiency, IgE (immunoglobulin E), intravenous immunoglobulin, omalizumab

## Abstract

Chronic spontaneous urtcaria (CSU) can represent the leading sign of a wide spectrum of systemic diseases, including primary immunodeficiencies. We describe the case of a young adult female with coexisting CSU and common variable immunodeficiency (CVID) successfully treated with omalizumab. The patient, with a history of recurrent respiratory infections during childhood, was referred to clinical attention due to the development of refractory CSU. During the diagnostic workup for the research of secondary causes of urticaria, an immunological assessment was performed, showing markedly reduced levels of IgG and IgM, poor antibody response against vaccinating antigens in absence of a T cellular deficiency. Therefore, the diagnosis of CVID was posed. Despite the immunoglobulin replacement and a trial with intravenous immunoglobulin at immunomodulatory dosage, the patient continued to experience severe urticaria, with significant impairment in the quality of life. After 2 years from the diagnosis of CVID, a treatment with omalizumab was started, showing complete remission of cutaneous symptoms after the first injection. The drug was well-tolerated, and the patient did not experience adverse effects during a 12-months follow-up.

## Introduction

Chronic spontaneous urticaria (CSU) is defined by the spontaneous appearance of wheals, angioedema or both for at least 6 weeks (1). The etiology of CSU is often referred as idiopathic, after having investigated the possible secondary causes. Interestingly, it can represent the first sign of a wide spectrum of systemic diseases, including primary and secondary immunodeficiencies and other conditions featured by dysregulation of the immune system or of the inflammatory response (2). Current management of CSU is organized into a step-care fashion, which includes the use of H1/H2-antihistamines as first step followed by immunosuppressive agents, such as cyclosporine or the anti-IgE monoclonal antibody omalizumab, for severe refractory cases (1). However, data on the efficacy and safety of omalizumab in patients with coexisting CSU and common variable immunodeficiency (CVID) are lacking because primary immunodeficiency diseases are usually excluded from clinical trials on biologics (3). Herein, we describe the first case of refractory CSU successfully treated with omalizumab in a young adult affected with CVID, after obtaining informed patient's consent.

## Case Report

We describe the case of a 19-year-old adult female with a history of recurrent upper respiratory tract infections since early childhood and a significant episode of pneumonia requiring prolonged hospitalization at 5 years of age. At 10 years of age, the patient started experiencing recurrent episodes of diffuse itching wheals, which were not apparently elicited by any physical triggers and were sometimes associated with cough and dyspnea. At 15 years of age, due to the worsening of the cutaneous symptoms, almost occurring daily and persisting for more than 6 weeks despite being treated with second-generation H1-antihistamines at 2-fold the approved doses, a full allergy diagnostic workup for CSU was performed: skin prick testing for food and aeroallergens, spirometry with bronchodilator response, a complete blood cell count with differential, C-reactive protein, serology for Helicobacter Pylori, complement fractions C3 and C4, antinucleus antibodies, thyroid hormones and auto-antibodies, celiac disease auto-antibodies, and hepatic and renal function produced negative or normal results.

**Figure 1:**
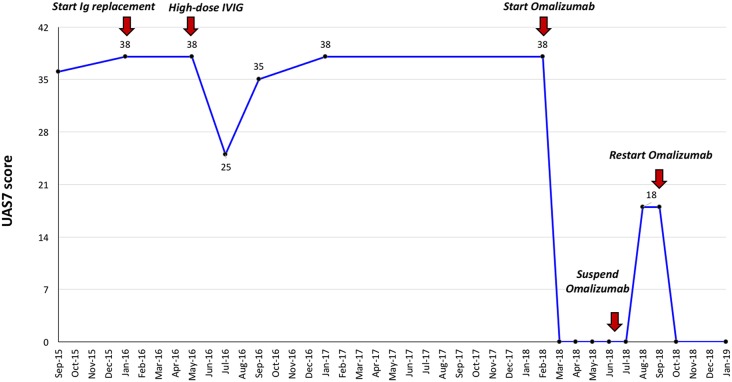
Effect of immunoglobulin replacement therapy and omalizumab on clinical severity of chronic urticaria by UAS7 score. Ig, Immunoglobulin; IVIG, intravenous immunoglobulin.

Given her history of recurrent respiratory infections, immunological exams were then performed, which showed a significant reduction in two serum immunoglobulin isotypes (IgG 449 mg/dL, −3 SD; IgM 64 mg/dL, −1 SD), low levels of B lymphocytes (58/mm^3^, <1% of total lymphocytes), poor specific humoral response against common vaccinating antigens (anti-tetanus IgG 0.10 IU/mL, protective response >1,00 IU/mL; anti-B hepatitis IgG 0,00 mIU/mL, protective response >10 mIU/mL) and no evidence of T cell deficiency (normal values of T lymphocytes subset and of T cell proliferation). Analysis for known genetic causes of hypogammaglobinemia (including BAFF, TACI, TNFRSF, and BTK mutations) proved negative. Therefore, considering the clinical phenotype (increased susceptibility to infections) the immunological features and the absence of other demonstrated causes of hypogammaglobulinemia, a diagnosis of common variable immunodeficiency (CVID) was made (4). Replacement therapy with subcutaneous human immunoglobulins (Ig) (0.55 g/kg/month) was started, resulting in a significant and persistent improvement of respiratory but not of cutaneous symptoms. Therefore, following the first 5 months of subcutaneous Ig replacement therapy, a trial with intravenous Ig (IVIG) at a higher immunomodulatory dose (0.8 g/kg/month) was initiated, resulting in moderate improvement in cutaneous symptoms, with a reduction in the Urticaria Activity Score over 7 days (UAS7) from 38 to 25/42 points. Nevertheless, after 3 cycles of high-dose IVIG, the patient had a recurrence of severe CSU and thereafter continued to experience uncontrolled cutaneous symptoms for 2 years, despite being concomitantly treated with different combinations of second-generation H1-antihistamines up to 2- to 3-fold the approved doses together with an H2-antihistamine and/or a leukotriene receptor antagonist (Figure 1). At the age of 17.8 years old, considering the substantial impact of CSU on everyday quality of life with an UAS7 of 38, and the recurrent need for short courses of oral prednisone, an add-on treatment with omalizumab was started, 300 mg every 4 weeks, resulting in complete remission of urticaria symptoms within a week from the first injection, with no further need for controller medications (Figure 1). After 6 injections, omalizumab was stopped for 2 months according to the therapeutic schedule approved in Italy, which caused a relapse of urticaria, albeit less severe (i.e., UAS7 of 18). She was restarted on omalizumab, which led to an immediate resolution of skin lesions (Figure 1). The patient continued treatment with subcutaneous Ig, and despite having received 12 injections of omalizumab so far, she has not reported having side-effects.

## Discussion

Urticaria can be the first manifestation of a wide spectrum of conditions involving the immune and inflammatory response. In this regard, although rare, particularly relevant is the association between urticaria and autoinflammatory diseases featured by alterations in the cryopririn pathway, as Cryopyrin-associated periodic syndromes (CAPS), including Familial cold urtcaria syndrome (FCAS), Muckle-Wells syndrome, and Chronic infantile neurologic cutaneous articular (CINCA) syndrome (5–7). Moreover, urticaria is part of the complex clinical phenotype of the recently described PLCG2-associated antibody deficiency and immune dysregulation (PLAID) (8).

The interest on the association between immunodeficiencies (ID), atopic disease and autoimmunity is currently increasing, since several studies (9–12), have shown that patients with primary ID, and particularly CVID, are a population at risk of developing a wide spectrum of autoimmune manifestations, with a predominance in adult females (13). Concerning CVID, despite the absence of predictor of autoimmunity, it has been shown that patients which develop autoimmunity often have a marked reduction of CD19 cells, as evidenced in our patient (14, 15). However, the presence of atopic diseases and urticaria, although reported in a considerable percentage of patients with IgA deficiency (16), is rarely described in patients with CVID (13, 17). Of note, some recent case reports, mainly including adult patients, have reported CSU as the first or the leading sign of CVID, similar to our case (17, 18).

The complex pathogenic mechanism underlying urticaria in CVID is not completely understood. In this setting, it has been hypothesized that CU could be elicited either by infections and consequent complement activation by inappropriate humoral response, or by the high susceptibility to autoimmune manifestations, due to the presence of antibody against the high affinity IgE receptors FcεRIα (that cause the cross-linking of adiacent receptors) or against IgE molecules, determining in both cases the degranulation of mast cells and basophils (19, 20).

IVIG replacement is the mainstay treatment for CVID as it restores antibody function (21). Additionally, IVIG therapy has potential anti-inflammatory and immunomodulatory activities that might reduce autoantibody-mediated inflammation (22, 23). As such, IVIG therapy has been shown to be effective in some patients with refractory CSU associated with autoimmunity and CVID (17). However, in our case, Ig administered at both substitutive and immunomodulatory dosages did not induce a complete resolution of CSU.

Omalizumab is a humanized monoclonal IgG1 anti-IgE antibody, which binds to free IgE and inhibits their interaction with the FcεRI receptors on mast cells and basophils, leading to a downregulation of such receptors (24). Omalizumab is recommended as a third-line treatment for refractory CSU (1), although its definitive mechanism of action in CSU is not currently elucidated and likely goes beyond the modulation of mast cell and basophil IgE receptors function (24). Indeed, a fixed dose of omalizumab is approved for all CSU patients, regardless of serum total IgE level, as opposed to the dosing needed for the treatment of severe asthma (24). In addition, recent evidence showed that the change in IgE level after the first injection of omalizumab could be the best predictor of response to this biological treatment, as all patients with low baseline IgE (i.e., <43 IU/ml) who at least doubled the total IgE levels before the second injection of omalizumab became complete or partial responders (25).

Available data on the efficacy and safety of omalizumab for CSU coexisting with immunodeficiency diseases are limited to a few case reports, mainly including patients with hyper-IgE syndrome and HIV infection. In particular, Bard et al. (26) reported that omalizumab was effective and safely used in a case of hyper-IgE syndrome presenting with severe eczema. Iemoli et al. (27) described the effective use of omalizumab for CU in a patient with HIV infection, without side-affects in terms of infections and interference with antiretroviral therapy.

Regarding safety, there is only one report of an adult female with Ig replacement therapy treated-CVID, who developed persistent elevation of peripheral blood myeloid cell counts after being treated for 29 months with omalizumab for concomitant severe asthma and who showed a prompt normalization of such counts after interrupting the biological therapy (28).

## Concluding Remarks

We describe the first case reporting the efficacy and the short-term safety of omalizumab for refractory CSU in an adolescent with CVID, suggesting a possible window of opportunity for the use this biologic agent in the immunosuppressed host. Extended follow-up studies are needed to address the long-term safety of omalizumab for the treatment of CSU in patients receiving Ig replacement therapy for CVID.

## Data Availability

The datasets generated for this study are available on request to the corresponding author.

## Ethics Statement

Written informed consent was obtained from the individual(s) for the publication of any potentially identifiable images or data included in this article.

## Author Contributions

PC, GC, NC, AL, and SD'E drafted the manuscript. PC, RC, and DP critically reviewed it for important intellectual contents. All authors gave the final approval of the version to be published.

## Conflict of Interest Statement

The authors declare that the research was conducted in the absence of any commercial or financial relationships that could be construed as a potential conflict of interest.
